# Occipitoparietal contributions to recognition memory: stimulus encoding prompted by verbal instructions and operant contingencies

**DOI:** 10.1186/1744-9081-3-44

**Published:** 2007-08-21

**Authors:** Michael W Schlund, Michael F Cataldo

**Affiliations:** 1 Department of Behavioral Psychology, Kennedy Krieger Institute, Baltimore MD, USA; 2 Department of Psychiatry and Behavioral Sciences, Johns Hopkins University School of Medicine, Baltimore MD, USA

## Abstract

**Background:**

Many human neuroimaging investigations on recognition memory employ verbal instructions to direct subject's attention to a stimulus attribute. But do the same or a similar neurophysiological process occur during nonverbal experiences, such as those involving contingency-shaped responses? Establishing the spatially distributed neural network underlying recognition memory for instructed stimuli and operant, contingency-shaped (i.e., discriminative) stimuli would extend the generality of contemporary domain-general views of recognition memory and clarify the involvement of declarative memory processes in human operant behavior.

**Methods:**

Fifteen healthy adults received equivalent amounts of exposure to three different stimulus sets prior to neuroimaging. Encoding of one stimulus set was prompted using instructions that emphasized memorizing stimuli (Instructed). In contrast, encoding of two additional stimulus sets was prompted using a GO/NO-GO operant task, in which contingencies shaped appropriate GO and NO-GO responding. During BOLD functional MRI, subjects completed two recognition tasks. One required passive viewing of stimuli. The second task required recognizing whether a presented stimulus was a GO/NO-GO stimulus, an Instructed stimulus, or novel (NEW) stimulus. Retrieval success related to recognition memory was isolated by contrasting activation from each stimulus set to a novel stimulus (i.e., an OLD > NEW contrast). To explore differences potentially related to source memory, separate contrasts were performed between stimulus sets.

**Results:**

No regions reached supralevel thresholds during the passive viewing task. However, a relatively similar set of regions was activated during active recognition regardless of the methods and included dorsolateral and ventrolateral prefrontal cortex, right inferior and posterior parietal regions and the occipitoparietal region, precuneus, lingual, fusiform gyri and cerebellum. Results also showed the magnitude of the functional response in the occipitoparietal region was inversely correlated with reaction times (RTs), such that the largest functional response and slowest RTs occurred to Instructed stimuli and the smallest functional response and fastest RTs occurred to GO stimuli, with effects to NO-GO stimuli intermediate. The inverse relation was also present bilaterally in the parahippocampus and hippocampus. Comparisons between stimulus sets also revealed regional differences potentially related to source memory.

**Conclusion:**

Recognition of stimuli previously associated with instructions and operant contingencies (i.e., discriminative stimuli) generally recruited similar inferior frontal and occipitoparietal regions and right posterior parietal cortex, with the right occipitoparietal region showing the largest effect. These findings suggest domain-general views of recognition memory may be applicable to understanding the neural correlates of control exerted by discriminative stimuli and suggest declarative memory processes are involved in human operant behavior.

## Background

Numerous functional magnetic resonance imaging (fMRI) studies on episodic and recognition memory for words, picture and sounds, consistently find brain activation in various portions of the lateral and posterior parietal regions, medial and inferior frontal regions and various medial temporal lobe structures [1-4]. The reliability of findings has encouraged the development of domain-general views of human recognition memory [5-7]. One common feature of many human neuroimaging studies is to give subjects verbal instructions to direct their attention to a specific stimulus attribute (e.g., perceptual, semantic, source, spatial), which then prompts encoding. Thus, instructions are a contextual variable that may be manipulated to lay the foundation for subsequent memory formation. The question addressed in this investigation is whether the same or a similar neurophysiological process is involved when the contextual variable prompting memory formation involves contingency-shaped, rather than instructed responses.

Converging evidence from a diverse number of investigations shows memory formation and subsequent recall/recognition is highly sensitive to contextual variables present during encoding, particularly reward. For instance, Wittmann et al. [8] examined long-term recall for object pictures and reported greater dopaminergic midbrain activation to items that predicted monetary rewards, reward associated items were recalled better, and reward-associated items elicited greater hippocampal activation. Adcock et al. [9] has shown that that long-term memory for scenes encoded along with monetary reward enhanced recall and were associated with greater activation during encoding. Visual cortex and parietal regions associated with the allocation of spatial attention in a visual cueing task also show enhancement by the presence of reward incentives for speeded performance [10]. Ramnani and Miall [11] also showed greater activation within the left parahippocampal gyrus when reward was present in a motor task.

This investigation examined the effects of three different methods of prompting encoding on activation during recognition memory. Encoding in one condition was prompted by verbal instructions. In the remaining conditions, encoding was prompted by trial and error learning within the context of an operant (instrumental) learning task. We hypothesized that during operant learning, the three-term contingency (i.e., stimulus-response-consequence relation) prompts encoding of a discriminative stimulus in ways that parallel verbal instructions. Accordingly, retrieval-based activation correlated with stimulus recognition should be relatively similar between instructed stimuli and discriminative stimuli and be localized in dorsolateral and ventrolateral prefrontal cortex and posterior parietal regions, including the precuneus, lingual gyrus, and particularly occipitoparietal regions [12-16]. Findings highlighting a common spatially distributed network during recognition memory of instructed and discriminative stimuli would facilitate the generality of domain-general theories of human memory functioning. Findings in line with our prediction would also reinforce and extend nonhuman based neurophysiological theories on the relation between operant behavior and declarative memory, which involves the conscious recollection of facts and events [17-20].

In the present investigation, encoding of stimulus information was prompted using three different methods prior to neuroimaging. Encoding of two stimulus sets was prompted by operant contingencies embedded within a GO/NO-GO task. Inclusion of the NO-GO condition, which includes a contingency but no reward delivery, provides a novel test of whether activation observed during recognition of GO stimuli is reward dependent. Encoding of the third stimulus set was prompted by verbal instructions that emphasized memorizing stimuli. During two separate functional neuroimaging runs, subjects completed a passive and an active recognition memory task, with task order counterbalanced across subjects. Both tasks presented individual stimuli from each stimulus set and an additional novel stimulus ('NEW') used as a baseline for the neuroimaging analysis. The passive memory task required only observation of stimuli. In contrast, the active recognition memory task required making a source or categorical judgment regarding whether a stimulus was a GO/NO-GO stimulus, an Instructed stimulus or a NEW stimulus. This methodology was adapted from episodic memory studies in which encoded stimuli, referred to as 'OLD', are contrasted with 'NEW' stimuli to identify activation related to 'retrieval success.' Potential differences related to source memory were examined by performing contrasts between stimulus sets. Finally, varying the response requirement (passive vs. active) between tasks enabled examination of the relation between regional activation and response dependency.

## Methods

Fifteen healthy, right-handed males (n = 8) and females (n = 7) participated. Subjects were Johns Hopkins employees, students, and Baltimore residents. All were unfamiliar with the task and reported being between 18 and 50 years of age, right-handed, free of medications affecting the central nervous system or the autonomic system for at least 2 weeks, and without a personal history of psychiatric disorder or a psychiatric history in first-degree relatives. Informed, written consent was obtained from all subjects according to the institutional guidelines established by the Johns Hopkins Human Subjects Protection Committee.

### Experimental conditions

Stimulus encoding occurred outside of the fMRI scanner approximately three hours before neuroimaging. Stimuli consisted of nine Greek letters (α, ∏, ∑, ⋂, μ, λ, δ, β, Ω), approximately 7.6 cm by 7.6 cm in size, that were randomly assigned to encoding conditions for each subject, thereby minimizing confounds related to stimulus features in the imaging analysis. Training took place in a quiet room with the subject seated in front of the computer and keyboard. There were three encoding conditions: GO, NO-GO and Instructed. GO and NO-GO encoding conditions occurred concurrently during operant training. The order of completing encoding conditions was counterbalanced across subjects, such that half received the Instructed encoding condition first, followed by operant training under GO and NO-GO encoding conditions

**Figure 1:**
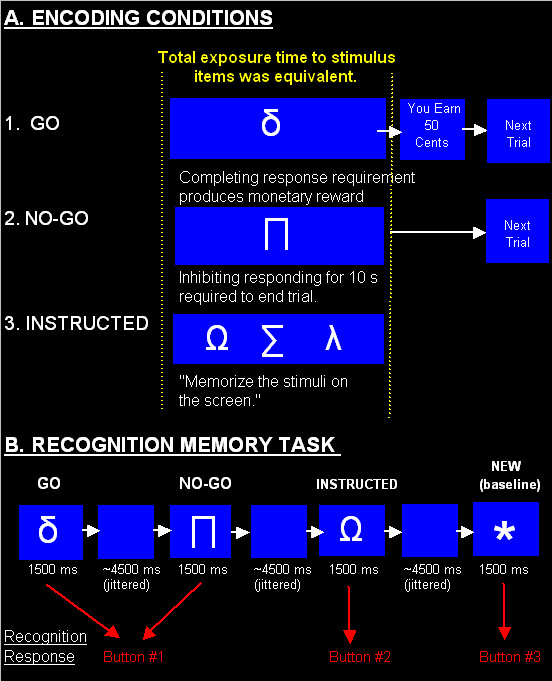
**Trial types used in each encoding condition prior to functional neuroimaging**. (A) During GO trials, completing a response requirement on a response button in the presence of GO stimuli produced money. During NO-GO trials, withholding responding in the presence of NO-GO stimuli for 10 s terminated the stimulus and trial. In the Instructed encoding condition, subjects were told to memorize a set of stimuli. Subjects received equivalent amounts of exposure to stimuli in all three encoding conditions prior to neuroimaging. (B) During the active recognition memory task completed during neuroimaging, stimuli from each encoding condition were presented in a random order along with a baseline stimulus (asterisk). The recognition response involved making a categorical judgment regarding whether a stimulus was observed in the GO/NO-GO training condition (button #1), the instructed condition (button #2), or was NEW (button #3).

The sequence of extended operant training followed by BOLD functional MRI was modeled after established procedures used in previous operant-fMRI investigations [for additional details see [21,22]]. At the start of training (i.e, encoding), task instructions emphasized that when a stimulus appeared on a computer screen pressing a designated response button would sometimes produce money, thus, it was up to subjects to choose when to press/not to press, earn as much money as possible and to pay careful attention to the stimuli as they would be presented later during neuroimaging. Instructions highlighting the future memory test served the function of encouraging similar levels of intentional encoding of stimulus information. Operant training consisted of learning two stimulus-response-consequence contingencies: GO and NO-GO. Three GO stimuli and three NO-GO stimuli were presented individually in a randomized order during training (e.g., GO, GO, NO-GO, GO, NO-GO, etc...). Panel A labeled "Encoding Conditions" in Figure 1 provides a schematic diagram for each contingency. For NO-GO stimuli, a period of 10 s without a response in the presence of the stimulus terminated the trial and initiated the next trial. For GO stimuli, reinforcers (either $0.05, $0.50 and $5.00) were delivered on a variable-ratio 3 reinforcement schedule for responding. After earning five consecutive reinforcers under a GO stimulus, the next stimulus in the order (GO or NO-GO) was presented. Total exposure to GO and NO-GO stimuli was found to not differ significantly during encoding, thus differences in viewing durations between GO and NO-GO conditions would not likely confound imaging results. Operant training continued until the total number of responses emitted in the presence of GO stimuli was greater than 90% of the total number of responses emitted during a session – thus, percent responses to NO-GO stimuli was less than 10% of the total responses emitted. During the Instructed encoding condition, three stimuli were printed on the computer screen for 6 min, as seen in Figure 1, panel A. Subjects were instructed to memorize the stimuli over the next 6 minutes and to pay careful attention because the stimuli would be presented later during imaging – intentional encoding of stimulus information. A paired two-tailed *t*-test was performed to determine whether differences existed in total duration of exposure to stimuli in the Instructed condition (group mean = 6 min (thus, SE = 0 s)) and discriminative stimuli (mean = 5 min 52 s (SE = 9 s)). Results showed no significant differences in exposure (*t *(14) = 2.02, *P *= 0.06). Thus, exposure to stimuli comprising Instructed, GO and NO-GO encoding conditions were similar, eliminating potential differences in viewing durations to stimuli during encoding as a potential confound in the imaging data.

### fMRI task and acquisition parameters

Neuroimaging occurred approximately 2–3 hours after training was completed. Subjects were placed in the scanner and handed a response box containing three response buttons arranged vertically. Instructions described the basic task and the function of each response button. Panel B in Figure 1 provides a schematic diagram of the recognition memory task used and trial timings. Using an event related design, individual stimuli from each encoding condition were randomly presented on 18 trials for 1500 ms followed by a blank screen averaging 4500 ms, which effectively 'jittered' stimulus presentations across time such that stimulus onsets were separated by an average of 6 s. Subjects completed a passive and an active recognition memory task, which were presented in a counterbalanced order across subjects. For the passive memory task, stimuli were presented and subjects were instructed to observe each stimulus and make no button presses. For the active recognition memory task, subjects were instructed to press the button #1 (top button) if the stimulus was seen during the behavioral (operant) training condition, button 2 (middle button) if the stimulus was seen during the Instructed training condition, and button 3 (bottom button) if the stimulus was novel (NEW). The NEW stimulus used was an asterisk and served as the baseline condition for performing conventional OLD > NEW imaging contrasts to highlight regional activation correlated with recognition memory.

Functional MRI images were obtained on a 3 T Philips MRI scanner. Eprime software controlled stimulus presentation and recorded timing data. Task instructions and stimuli were presented on a rear screen monitor viewed through a mirror anchored to a standard head coil. After an initial series of sagittal T1-weighted localizers, a set of oblique T1-weighted images, angled parallel to the intercommissural line, were gathered. The fMRI data were acquired at the same slice locations. The T1 parameters were repetition time (TR) of 500 ms and an estimation time (TE) of 11 ms. Functional MRI data were gathered using a single-shot echo planar imaging (EPI) sequence for data acquisition, with a TR of 2000 ms, a TE of 50 ms, and a 90-degree flip angle. The matrix size was 64 × 64 and the field of view 24 cm, yielding voxels measuring 3.75 × 3.75 mm in plane. Using these parameters, 43 contiguous slices were obtained angled parallel to the intercommissural line.

### fMRI analyses

For a subject's imaging data to be included in the analysis, head movement was limited to less than 2 mm. All preprocessing and data analysis were performed using statistical parametric mapping software, version 2 [23]. EPI images were slice-timing corrected to adjust for the lag between slices during each TR, corrected for head motion during scanning, and normalized to a standard template brain from the Montreal Neurological Institute (MNI) to get all participants into the same space [24]. After normalization, voxels were resampled with a 2 × 2 × 2 mm voxel size. EPI images were then spatially smoothed using a 6 mm full-width-half-maximum(FWHM) Gaussian kernel. High pass filtering was applied to the time series of EPI images to remove the low frequency drift in EPI signal and then subjected to a two-level analysis. At the first level, individual-subject models were constructed in which a linear regression analysis was performed between the observed event related EPI signals and onset times of stimuli (GO, NO-GO, Instructed and NEW) [25]. Contrast images were then produced by performing voxel-wise comparisons for stimuli within each encoding condition (i.e., OLD) relative to the baseline stimulus (i.e., NEW). Contrast images were analyzed at the second 'random effects' level using one-sample t-tests, for revealing the main effect of recognition and condition-specific activation, and multiple regression (simple correlation), for revealing linear increases in activation across conditions [26]. The thresholds *P *< .001, uncorrected for multiple comparisons, and 20 contiguous voxels were used except where noted. Analyses of medial temporal regions were performed using separate anatomically defined masks, which employs a small volume correction, created with the Wake Forest University PickAtlas SPM2 plug-in [27]. The location of voxels with significant activation was summarized by their local maxima separated by at least 8 mm, and by converting the maxima coordinates from MNI to Talairach coordinate space using linear transformations [28]. These coordinates were finally assigned neuroanatomic labels using human brain atlas' and the Talairach Daemon [29].

## Results

### Behavioral

**Figure 2:**
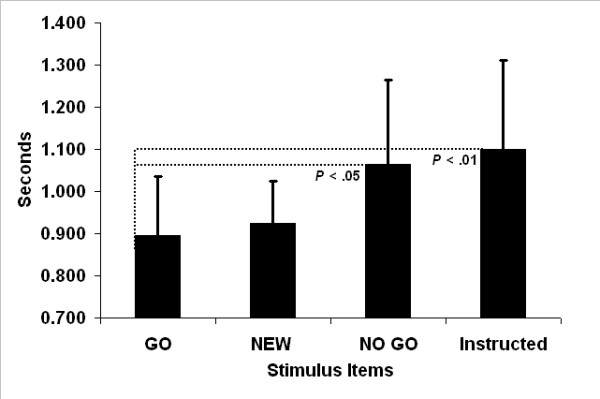
**Reaction times during recognition memory**. Group mean reaction times and standard deviations of recognition memory judgments exhibited during neuroimaging to stimulus items with different encoding histories. Encoding occurred prior to imaging and was prompted by three conditions (1) GO stimulus items: pressing a button to earn money under one set of stimuli; (2) NO-GO stimulus items: inhibiting button pressing under a second set of stimuli; and (3) Instructed stimulus items: memorizing a third set of stimuli as prompted by verbal instructions. The item labeled "NEW" was an asterisk presented during neuroimaging that prompted a button press and functioned as the baseline condition for assessing activation correlated with the onsets of encoded stimulus items (i.e., OLD > NEW contrasts). Significant differences in reaction times were observed between GO and NO-GO conditions and GO and Instructed conditions.

All subjects responded with 100% accuracy during the active recognition memory task. Paired *t*-test analyses were used to compare differences in reaction times among GO, NO-GO, Instructed and the NEW baseline condition. Group mean reaction times and standard deviations appear in Figure 2. Reaction times for GO stimuli were found to be significantly faster than NO-GO ((*t *(14) = 2.9, *P *= 0.0117) and Instructed stimuli (*t *(14) = 4.01, *P *= 0.0013), but not NEW stimuli (*t *(14) = .55, *P *= 0.591). All other comparisons did not reach significance, however, reaction times for NEW stimuli relative to Instructed stimuli did approach significance (*t *(14) = 1.97, *P *= 0.069).

### Neuroimaging

#### Main effect for recognition

**Figure 3:**
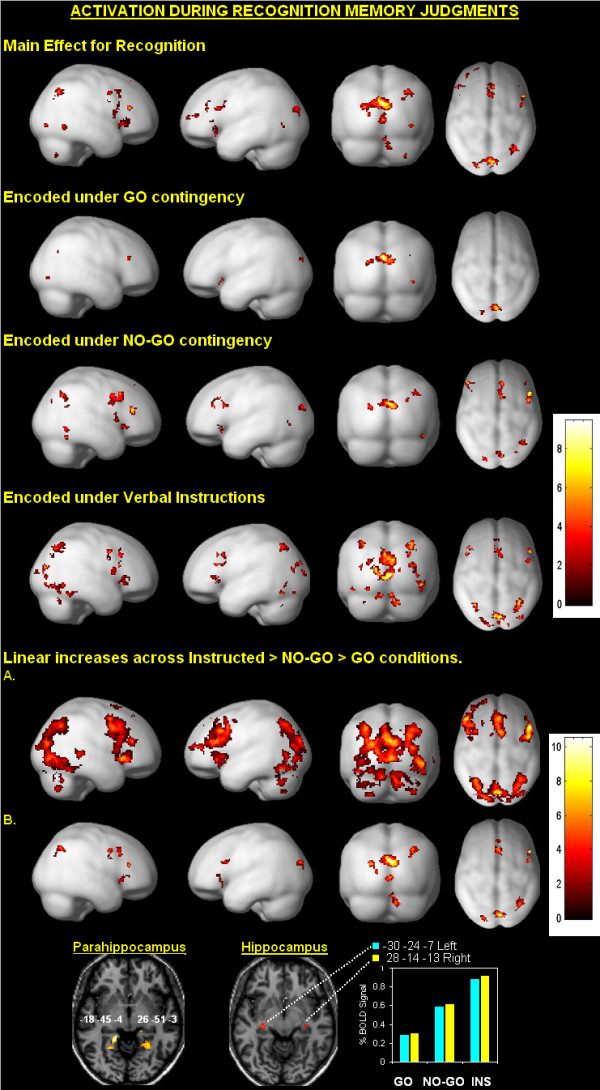
**Activation correlated with recognition memory by condition**. The top row shows regional activation for the main effect of recognition relative to baseline (i.e, the NEW stimulus). Subsequent rows reveal regional activation during recognition of GO, NO-GO and Instructed (INS) stimulus items relative to a baseline. The most prominent and consistently activated region across conditions was the occipitoparietal region. Activation was also noted in varying degrees in dorsolateral and ventrolateral prefrontal cortex and posterior parietal regions. Linear increases in activation reflecting magnitude differences across conditions were also noted (Instructed > NO-GO > GO). The regional increases were centered in the precuneus, superior and inferior frontal gyrus and right superior parietal regions (results shown in panel **A**. at *P *< .001 and in panel **B**. at *P *< .000001). The insert shows bilateral increases in the posterior parahippocampus and hippocampus.

For the passive viewing task, voxel-wise comparisons revealed no regions that exceeded our thresholds. For the active recognition memory task, Figure 3 (row 1) and Table 1 present results for the main effect of recognition memory (collapsed across encoding conditions) contrasted against activation to the NEW stimulus. Bilateral activation was observed in inferior, middle, and superior frontal regions and posterior parietal regions that included the precuneus and cuneus localized near the occipitoparietal sulcus. Additional activation was noted in the left cingulate gyrus, medial frontal gyrus and fusiform gyrus and right lingual gyrus, middle occipital gyrus, inferior and superior parietal regions and cerebellum.

**Table 1:** Regional activation for main effect of recognition.

					Peak	
Region		X	Y	Z	*t*	BA
Left	Inferior Frontal Gyrus	-30	21	-11	5.86	
	Medial Frontal Gyrus	-4	29	37	4.68	6
	Middle Frontal Gyrus	-51	34	20	4.26	
	Superior Frontal Gyrus	-20	59	6	5.78	
	Cingulate Gyrus	-12	-55	29	5.82	31
	Precuneus	-24	-71	20	5.5	
	Cuneus	-16	-64	9	6.18	
	Fusiform Gyrus	-26	-55	-9	4.8	
Right	Inferior Frontal Gyrus	38	21	-3	6.07	
	Superior Frontal Gyrus	4	33	48	5.82	8
	Middle Frontal Gyrus	50	16	40	4.52	8
	Lingual Gyrus	16	-66	5	9.39	
	Precuneus	10	-71	26	8.76	
	Cuneus	4	-81	8	5	17
	Middle Occipital Gyrus	40	-80	1	5.45	
	Inferior-Superior Parietal Lobule	32	-56	40	7.39	
	Declive	4	-76	-15	6.37	
	Medial Temporal Gyrus	46	-55	-2	5.96	

#### Condition effects

Voxel-wise contrasts comparing stimuli within each encoding condition to the NEW stimulus revealed no regions that exceeded our thresholds during the passive viewing task. For the active recognition memory task, subsequent rows in Figure 3 (rows 2–4) highlight regional activation for each encoding condition relative to the NEW stimulus, with complete results summarized in Table 2. Across encoding conditions there was considerable overlap in the localization of activation, as well as notable magnitude effects – discussed below. Stimulus onsets elicited bilateral activation in posterior parietal regions centered near the occipitoparietal sulcus, with the effect larger in the right cerebrum. Moreover, activation was generally localized in inferior and middle/medial frontal regions and the right inferior and superior parietal cortex.

**Table 2:** Regional activation during recognition for each encoding condition.

					Peak	
Contrast and Region		X	Y	Z	*t*	BA
GO > NEW						
Left	Inferior Frontal Gyrus	-36	17	-6	4.74	47
	Cingulate Gyrus	-10	-53	27	4.44	
	Precuneus	-22	-73	20	5.88	31
	Cuneus	-20	-86	25	4.61	18
	Cuneus	-14	-64	9	4.51	
Right	Medial Frontal Gyrus	36	30	19	5.16	
	Posterior Cingulate	8	-67	11	5.36	30
	Cuneus	10	-76	26	6.97	18
	Middle Occipital Gyrus	40	-78	1	5.24	
	Lingual Gyrus	14	-66	-2	4.67	18
	Medial Temporal Gyrus	36	-63	29	5.18	
NO-GO > NEW						
Left	Middle Frontal Gyrus	-51	34	20	5.50	
	Medial Frontal Gyrus	-6	44	16	5.23	9
	Inferior Frontal Gyrus	-32	25	-3	4.98	
	Parahippocampus	-26	-45	-10	6.89	37
	Precuneus	0	-72	29	6.16	
	Fusiform Gyrus	-26	-53	-7	5.00	
	Cuneus	-24	-82	24	4.90	
	Tonsil	-12	-48	-31	7.40	
	Culmen	-2	-55	-16	4.36	
Right	Inferior Frontal Gyrus	38	32	15	7.87	
	Superior Frontal Gyrus	2	14	56	5.51	
	Middle Frontal Gyrus	48	8	36	5.47	9
	Medial Frontal Gyrus	2	20	45	4.48	
	Precentral Gyrus	53	10	12	4.56	44
	Frontal-Temporal	55	12	3	4.65	
	Lingual Gyrus	16	-66	5	8.65	
	Precuneus	10	-73	26	8.47	
	Cuneus	26	-74	31	4.46	
	Inferior Parietal Lobule	36	-54	38	6.36	
	Fusiform Gyrus	48	-57	-12	6.72	37
	Medial Temporal Gyrus	34	-61	27	6.01	
	Superior Temporal Gyrus	44	-53	25	5.37	39
Instructed > NEW						
Left	Inferior Frontal Gyrus	-36	29	4	7.72	
	Middle Frontal Gyrus	-50	17	34	5.72	
	Posterior Cingulate	-16	-60	10	5.56	
	Middle Occipital Gyrus	-48	-65	-10	5.88	37
	Cuneus	-24	-82	26	5.12	
	Lingual	-12	-49	1	4.56	19
	Superior Parietal Lobule	-36	-60	49	5.71	
	Inferior Parietal Lobule	-36	-58	42	4.18	
	Declive	-6	-76	-15	4.05	
	Uvula	-30	-65	-25	4.27	
	Insula	-40	19	0	4.89	13
	Medial Temporal Gyrus	-36	-77	19	5.11	
Right	Inferior Frontal Gyrus	53	12	12	5.88	44
	Middle Frontal Gyrus	50	6	37	5.64	9
	Medial Frontal Gyrus	6	16	47	5.43	6
	Cingulate Gyrus	8	23	39	4.26	
	Cuneus	6	-80	32	8.22	19
	Lingual Gyrus	16	-62	0	7.57	19
	Middle Occipital Gyrus	48	-74	-3	5.61	
	Inferior Parietal Lobule	32	-56	43	9.21	
	Superior Parietal Lobule	34	-64	44	5.00	7
	Declive	28	-63	-17	7.60	
	Uvula	34	-63	-25	4.62	
	Pyramis	10	-75	-27	6.44	
	Insula	38	21	1	4.93	
	Fusiform Gyrus	50	-41	-11	4.55	37

#### Magnitude effect

Given the orderly increases in reactions times observed across conditions (i.e., Instructed > NO-GO > GO) coupled with the observation of increases in the extent of activation across encoding conditions in Figure 3, we performed a simple regression analysis to localize linear increases in activation across encoding conditions (i.e., Instructed > NO-GO > GO) with special attention given to occipitoparietal regions. The analysis used both *P *< .001 and *P *< .000001 thresholds, uncorrected for multiple comparisons, and an extent threshold of 20 contiguous voxels. Results presented in Figure 3 (rows 5 and 6) and Table 3 highlight regions showing significant increases in activation across encoding conditions. The primary finding was activation centered near the occipitoparietal sulcus, with the extent of activation more extensive in the right cerebrum. Linear bilateral increases in activation were also observed in inferior and precentral frontal regions, cuneus, middle occipital gyrus and the superior parietal region. Activation was also noted in the left medial frontal gyrus and right middle and superior gyrus, lingual gyrus, precuneus and cerebellum. Results in Figure 3 also show at reduced statistical thresholds bilateral increases in activation in the posterior parahippocampus and hippocampus (parahippocampus: maxima = *P *< .001; left, *t *= 5.33, right *t *= 3.93). Plots of percent signal change for the hippocampus also highlight the linear effect (left maxima at *P *= .011, *t *= 2.36, and right maxima at *P *= .027, *t *= 1.97).

**Table 3:** Linear increases in activation: Instructed > NO-GO > GO.

					Peak	
Region		X	Y	Z	*t*	BA
Left	Inferior Frontal Gyrus	-34	27	4	7.82	
	Medial Frontal Gyrus	-4	27	37	6.00	
	Precentral Gyrus	-42	25	34	6.27	9
	Cuneus	-24	-80	24	7.28	
	Middle Occipital Gyrus	-18	-87	15	6.36	18
	Superior Parietal Lobule	-32	-70	46	6.23	
Right	Medial Frontal Gyrus	40	32	17	7.29	
	Inferior Frontal Gyrus	38	21	-3	7.12	
	Middle Frontal Gyrus	50	8	38	6.89	
	Precentral Gyrus	42	1	26	6.85	6
	Superior Frontal Gyrus	4	14	56	6.28	6
	Lingual Gyrus	16	-66	5	10.49	
	Cuneus	8	-68	7	8.20	
	Precuneus	10	-71	26	9.35	
	Cuneus	8	-78	24	9.33	
	Middle Occipital Gyrus	34	-81	21	6.52	19
	Superior Parietal Lobule	36	-62	44	6.26	7
	Precuneus	32	-66	35	6.24	
	Declive	4	-76	-15	7.19	
	Pyramis	10	-75	-23	6.62	
	Inferior Temporal Gyrus	46	-58	-2	7.31	

#### Source memory contrasts

Contrasts performed among the encoding conditions provides a means of exploring differences in activation during recognition memory that might vary as a function of different methods or sources that prompted encoding of stimulus information. Results highlighting voxel-wise differences between selected encoding conditions appear in Table 4 (*P *< .005, uncorrected for multiple comparisons, using an extent threshold of 20 contiguous voxels). Contrasting *GO > Instructed *revealed bilateral activation primarily in the insula and claustrum, regions with ties to affective processing and reward processing, as well as the right cingulate and superior temporal gyrus. Contrasting *NO-GO > Instructed *evidenced greater bilateral activation in the supramarginal gyrus and parahippocampus and the left precentral gyrus and insula and right superior and medial frontal gyrus, cingulate and fusiform gyrus. For the *GO > NO-GO *contrast, GO stimuli elicited activation in the precuneus. Contrasting *NO-GO > GO *revealed bilateral activation in middle frontal and precentral cortices, cingulate, lateral posterior nucleus of the thalamus, fusiform gyrus and precuneus. Right localized activation occurred in the medial, superior and postcentral frontal gyri, supramarginal and angular gyri and the inferior parietal lobule. Regional activation was also observed in the left posterior cingulate, insula and inferior temporal gyrus.

**Table 4:** Regional activation for contrasts between encoding conditions.

					Peak	
Contrast and Region		X	Y	Z	*t*	BA
GO > Instructed						
Left	Insula	-42	-19	5	5.28	13
	Claustrum	-34	-15	6	4.38	
Right	Insula	32	-24	21	6.91	
	Cingulate	8	-10	36	4.88	
	Superior Temporal Gyrus	48	-42	15	4.51	
	Precuneus	22	-51	30	4.13	
GO > NO-GO						
Right	Precuneus	20	-57	32	3.75	
NO-GO > Instructed						
Left	Precentral Gyrus	-44	-6	26	3.49	
	Parahippocampus	-22	-17	-23	4.5	
	Insula	-28	-32	18	5.6	
	Supramarginal Gyrus	-44	-49	30	4.34	
Right	Superior Frontal Gyrus	10	56	32	4.07	9
	Medial Frontal Gyrus	10	48	34	3.32	
	Cingulate	8	-41	33	5.59	
	Parahippocampus	38	-43	-6	3.41	
	Fusiform Gyrus	34	-41	-13	4.58	
	Supramarginal Gyrus	46	-53	27	3.46	
NO-GO > GO						
Left	Middle Frontal Gyrus	-46	12	38	4.22	8
	Precentral Gyrus	-42	17	34	3.56	9
	Cingulate	-8	12	38	3.92	
	Posterior Cingulate	-4	-38	24	3.24	23
	Thalamus: Lat Post Nuc.	-20	-21	14	4.51	
	Insula	-32	-28	16	3.70	13
	Inferior Temporal Gyrus	-48	-66	-2	5.54	
	Fusiform Gyrus	-44	-49	-16	3.94	
	Precuneus	-12	-48	47	4.51	7
Right	Medial Frontal Gyrus	8	29	43	6.48	8
	Middle Frontal Gyrus	26	16	42	6.83	
	Precentral Gyrus	48	1	26	5.10	6
	Superior Frontal Gyrus	24	48	23	4.71	
	Thalamus: Lat Post Nuc.	20	-19	16	5.26	
	Cingulate	14	9	35	6.20	32
	Fusiform Gyrus	50	-45	-15	4.28	37
	Supramarginal Gyrus	46	-37	33	5.39	
	Angular Gyrus	40	-55	34	4.55	
	Inferior Parietal Lobule	42	-52	43	4.50	40
	Precuneus	8	-62	38	4.01	7
	Postcentral Gyrus	40	-29	51	3.56	

## Discussion

In summary, the present investigation yielded three major findings of importance to human neuroimaging investigations on memory and human operant behavior. First, voxel-wise contrasts revealed no regions that reached statistical significance during the passive viewing task, which suggests simple presentations of OLD stimuli is not sufficient to elicit significant activation. However, activation during active recognition was present bilaterally in inferior, middle, and superior frontal regions and posterior parietal regions that included the precuenus and cuneus localized near the occipitoparietal sulcus. Second, the localization of activation during recognition memory was found to be relatively similar for stimuli encoded under conditions involving verbal instructions and conditions involving operant contingencies. Each type of encoded stimulus set elicited bilateral activation near the occipitoparietal sulcus, with the effect larger in the right cerebrum. Moreover, activation was observed in inferior and middle/medial frontal regions and the right inferior and superior parietal cortex. Third, the magnitude of the functional response in the occipitoparietal region was also found to be inversely correlated with reaction times (RTs), such that the largest functional response and slowest RTs occurred to Instructed stimuli and the smallest functional response and fastest RTs occurred to GO stimuli, with effects to NO-GO stimuli intermediate. The inverse relation was also present bilaterally in the parahippocampus and hippocampus. Linear bilateral increases in activation were also observed in inferior and precentral frontal regions, cuneus, middle occipital and the superior parietal region.

Differences in the localization of activation between conditions and the linear increases in activation across conditions suggests the methods used to direct attention and prompt encoding of stimulus information may modulate activation during recognition. Several prior investigations have shown that elaborating on stimulus meaning during encoding facilitates subsequent recognition and retrieval. This "levels of processing" view suggests that stimuli encoded under operant contingencies may be more elaborately (deeply) processed because of the demands associated with trial and error learning, that is, forming the relations among the stimulus, response and the consequence. Accordingly, discriminative stimuli might be expected to be easily recognized and, therefore, be associated with *faster reaction times and greater activation *compared to stimuli encoded under instructions, which presumably may be processed on a more superficial level (shallow encoding) [e.g., [30]]. Naturally, this fundamental idea may not be limited to tasks involving operant contingencies, but rather extend to conditions that require extensive interactions with stimuli. However, while the level of processing idea is consistent with our observation of significantly faster reaction times for GO and NO-GO stimuli relative to Instructed stimuli, this view does not account for our findings that showed greater activation for Instructed stimuli relative to GO an NO-GO stimuli, particularly in the occipitoparietal region.

An alternative account suggested by findings from verbal working memory studies, in which slow reaction times were accompanied by a large functional response in posterior parietal cortex, is that recognition of Instructed stimuli was relatively more 'difficult' [14,15]. Related to the present findings, the slower reactions times to Instructed stimuli, and the larger functional response suggests these effects were a function of either differences in the methods that prompted encoding (instructed versus contingency shaped) or some other aspect of our procedure. Since the order of exposure to conditions was counterbalanced across subjects and the duration of exposure to stimuli across conditions was similar, the linear magnitude effect seems unrelated to these factors. One procedural difference that may have influenced the linear magnitude effect was presenting Instructed stimuli as a group and presenting discriminative stimuli individually. However, it is difficult to develop a plausible account of how differences in presentation would produce the linear increases observed. It seems more likely that once the operant contingencies (GO or NO-GO) exerted firm control over behavior, recognition required fewer resources, which resulted in less activation. By comparison, encoding of stimulus information prompted by verbal instructions required much less behavioral involvement. These differences in behavioral control or involvement produced by either operant contingencies or simply task involvement may be responsible for the magnitude effect observed. Accordingly, this view predicts greater levels of behavioral control or involvement prompted by a procedure would result in easier recognition, faster reaction times, and less activation in the occipitoparietal region.

Collectively, the present findings support our prediction that during operant or instrumental learning, the operant contingencies functioned in much the same way as verbal instructions by eliciting a relatively similar set of 'retrieval success' regions, with the largest effects observed in the occipitoparietal region. These results provide some preliminary support for extending domain-general theories of human recognition memory, based largely on pictures, words and sounds, and encoding prompted by verbal instructions [5-7], to stimuli encoded through nonverbal, goal-directed experiences involving operant learning processes. Because recognition memory is considered a declarative memory process requiring conscious recollection of stimulus information, the observation of similar activation patterns to Instructed and discriminative stimuli suggests similar neural process are engaged during memory retrieval and situations involving discriminative stimulus control.

## Conclusion

Our investigation examined activation during recognition memory to nonverbal stimuli previously encoded under operant learning contingencies and nonverbal stimuli encoded under verbal instructions. Results showed a relatively similar spatially distributed network was activated during active recognition, especially in the occipitoparietal region, but the magnitude of the functional response was modulated by conditions present during encoding. In general, the present findings suggest domain-general views regarding the neural correlates of recognition memory may be relevant to understanding operant behavior and offer additional support for operant learning as involving declarative memory. At a broader level, neuroimaging investigations on human memory systems that employ both conventional human imaging research procedures (i.e., instructed encoding) and nonhuman research procedures (i.e. operant learning paradigms) provide a novel context in which to investigate cross-species functional-anatomical similarities.

## Competing interests

The author(s) declare that they have no competing interests.

## Authors' contributions

MS and MC were responsible for the fMRI design and writing of the manuscript. MS carried out the data acquisition and analyses. Both authors read and approved the final manuscript.
